# Brachysyndactyly in Poland Syndrome

**DOI:** 10.7759/cureus.9755

**Published:** 2020-08-15

**Authors:** Pratyush Shahi, Apoorv Sehgal, Ahmer Zafar, Aarushi Sudan, Vishali Moond

**Affiliations:** 1 Orthopaedics, University College of Medical Sciences, Delhi, IND; 2 Internal Medicine, University College of Medical Sciences, Delhi, IND

**Keywords:** poland syndrome, brachysyndactyly, left-sided, reconstructive surgery, associated anomalies

## Abstract

A 36-year-old man presented with incidental findings of an asymmetric chest with hypoplastic and flattened left anterior chest wall due to absent left pectoralis major. He also had short and webbed fingers in the left hand. These deformities were present since birth. Chest X-ray showed hyperlucency on the left side. Computerized tomography (CT) scan showed an absence of the left pectoralis major. X-ray of the left hand showed hypoplasia of the proximal phalanx and aplasia of the middle and distal phalanges of the second digit, and aplasia of the middle phalanges of the third and fourth digits. A diagnosis of left-sided Poland syndrome with associated ipsilateral brachysyndactyly, which is a very rare entity, was made. The patient opted against any reconstructive procedure as he had a minimal functional limitation.

## Introduction

Poland syndrome is a rare congenital anomaly characterized by the absence of the sternocostal bundle of the pectoralis major muscle. It involves the right side in 75% of the cases and is frequently associated with other skeletal and visceral anomalies [1].

We, through this case report, aim to increase awareness about this rare disease and highlight the importance of proper workup to rule out the associated musculoskeletal and visceral anomalies, and the indications and modalities of reconstructive surgery. We also state that the presence of congenital hand deformity should prompt a search for Poland syndrome in the patient.

## Case presentation

**Figure 1:**
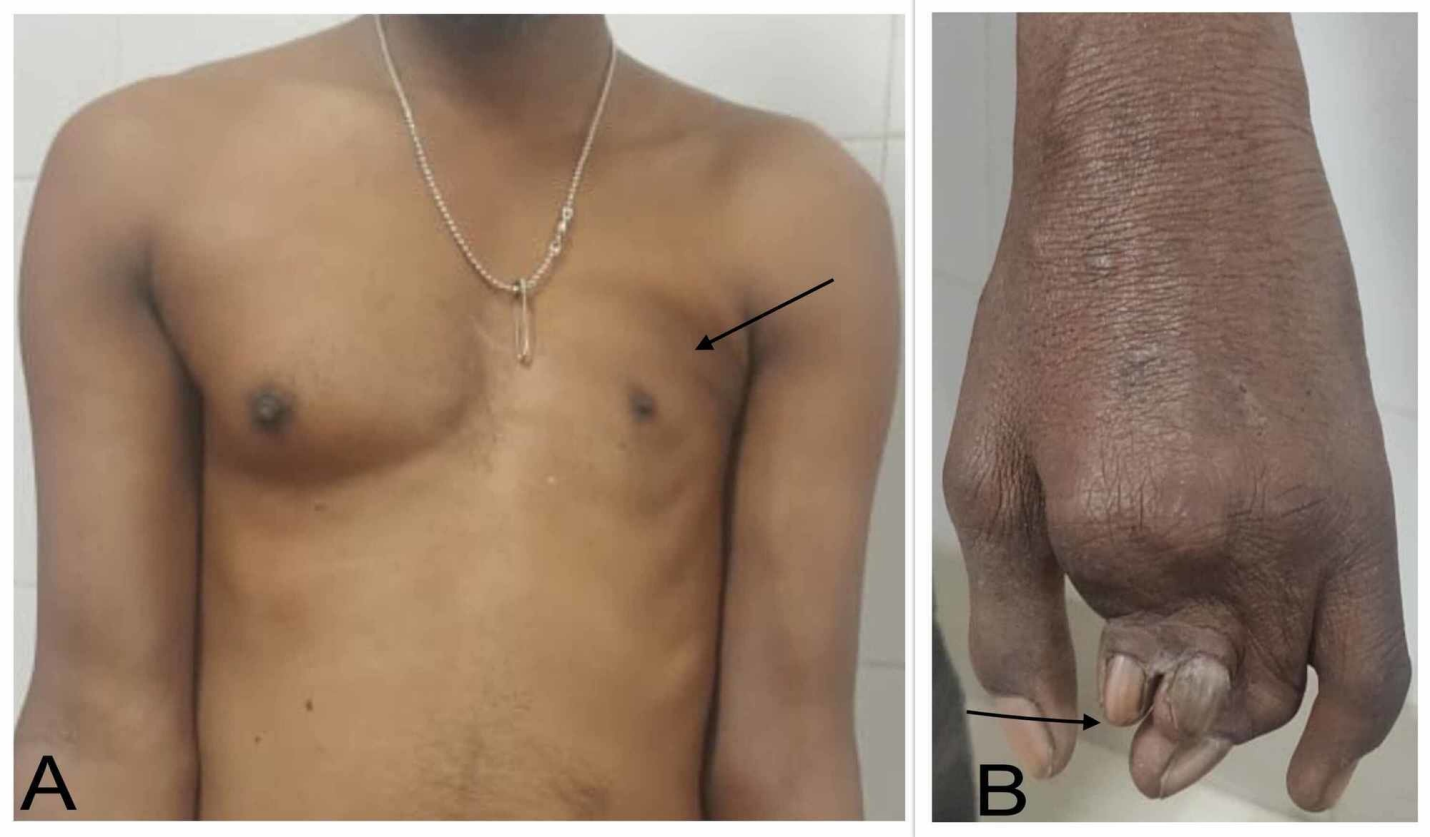
Clinical picture (A) Asymmetric chest with hypoplastic and flattened left anterior chest wall, absent pectoralis major, and smaller and higher left nipple; (B) Small and webbed 2nd, 3rd, and 4th digits representing brachysyndactyly of the left hand.

A 36-year-old man presented with cough for three days. Examination revealed incidental findings of an asymmetric chest with hypoplastic and flattened left anterior chest wall. There was an absence of the pectoralis major, axillary fold, and axillary hair on the left side (Figure 1A). The nipple on the left side was smaller and higher. Movements at the left shoulder were full. The patient also had short and webbed fingers (brachysyndactyly) in the left hand (Figure 1B). These deformities had been present since birth. Heart, breath, and bowel sounds were normal. The examination of the neck and back was unremarkable.

**Figure 2:**
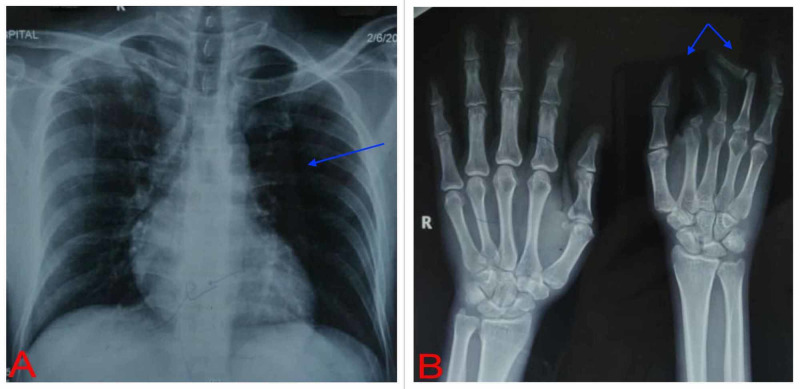
Radiographs (A) Chest X-ray showing hyperlucency on the left side with normal lung parenchyma, heart shadow, and ribs; (B) X-ray of the left hand showing hypoplasia of the proximal phalanx, aplasia of the middle and distal phalanges of the 2nd digit, and aplasia of the middle phalanges of the 3rd and 4th digits.

X-ray of the chest showed hyperlucency on the left side, with normal lung parenchyma, heart shadow, and ribs (Figure 2A). Computed tomography (CT) scan of the chest showed an absent left pectoralis major. X-ray of the left hand showed hypoplasia of the proximal phalanx, aplasia of the middle and distal phalanges of the second digit, and aplasia of the middle phalanges of the third and fourth digits (Figure 2B). Ultrasonography (USG) of the abdomen and pelvis was normal. All laboratory investigations were normal.

A diagnosis of left-sided Poland syndrome with associated brachysyndactyly was made. Options of available reconstructive procedures, along with the risks and benefits, were explained to the patient. He opted against it, as he had limited and manageable functional limitations and was not concerned about cosmesis.

## Discussion

Poland syndrome was first described by Alfred Poland in 1840 in a 27-year-old patient with complete unilateral absence of the sternal head of the pectoralis major and ipsilateral symbrachydactyly [2]. It is a rare congenital anomaly characterized by the unilateral absence or underdevelopment of pectoralis major and associated with involvement of adjacent shoulder muscles, rib abnormalities, dextrocardia, and ipsilateral hand deformities [3]. It has an incidence of 3-16 per 100,000 population and is more common in males [4]. The left side of the body is involved in only 25% and hand deformities are present in only 12% of the cases [5]. This is only the fourth case report of left-sided Poland syndrome reported from India; features of the previous three have been summarized in Table 1 [6-8].

**Table 1:** Cases of left-sided Poland syndrome reported from India

Author	Age of the patient	Associated features
Sunitha VC (2013) [6]	10 years	Hypoplastic left lung and left upper limb, dextrocardia, left-sided diaphragmatic hernia, spina bifida
Sharma CM (2014) [7]	8 years	Left-sided brachysyndactyly
Rajawat GS (2018) [8]	13 years	Left-sided brachysyndactyly, dextrocardia
Current case report	36 years	Left-sided brachysyndactyly

Poland syndrome is mostly sporadic and is thought to be caused due to a decreased thoracic blood supply on the affected side. Generally, an interruption of the embryonic blood supply in the subclavian artery is seen, which is inherited as an autosomal dominant trait [9]. Poland syndrome has been linked to 10p13-14 duplication and congenital hyperinsulinemia [10]. It has also been reported in association with a de novo deletion of 11q12.3 in monozygotic twins [11]. However, the underlying genetic etiology of Poland syndrome is still not established.

Becker’s nevus syndrome is a genodermatosis characterized by a cutaneous hamartoma (Becker’s nevus), which can be associated with cardiomyopathy, developmental delay, mental retardation, musculoskeletal abnormalities, and unilateral breast hypoplasia. Since the musculoskeletal anomalies in Poland syndrome are similar to those found in Becker's nevus syndrome, investigation regarding postzygotic mutations in beta-actin had been suggested by Cohen PR to study the potential relation between Poland syndrome and Becker's nevus syndrome [12].

Small and high scapula (Sprengel deformity), webbed neck with restricted movements (Klippel-Feil syndrome), scoliosis, paralysis of multiple cranial nerves (Moebius syndrome), craniofacial deformities, visceral anomalies like renal agenesis, hernia, and atrial septal defect, and blood dyscrasias like leukemia and lymphoma may be seen in association with Poland syndrome [13]. Dermatological manifestations like café-au-lait spots, cutaneous diffuse neurofibroma, acquired perforating dermatosis, congenital melanocytic nevus, congenital hemangioma, and psoriasis vulgaris have also been reported [14]. Hence, proper workup should be done to rule out the associated anomalies. The presence of congenital hand deformity should prompt a search for Poland syndrome in the patient.

Differential diagnoses of Poland syndrome include anterior thoracic hypoplasia (hypoplasia of the ipsilateral breast and superior location of the nipple-areola complex), Amazon syndrome (Poland syndrome associated with hypoplasia of the breast), and Klippel-Feil anomaly (failure of segmentation of cervical vertebrae leading to a short neck, restricted neck movements, and low posterior hairline) [6].

Treatment should be individualized depending on age, sex, degree of deformity, and patient’s preference [15]. Functional disability is minimal and the patient generally seeks surgery for cosmetic reasons. Other indications for surgery include paroxysmal movements of the chest wall and progressive lung herniation. Several reconstructive procedures have been described to correct the functional and structural deformities of the chest such as flaps (latissimus dorsi, rectus abdominis, and omental), lipofilling, and custom-made silicone prosthesis [16]. The syndactyly of Poland syndrome is usually the first component to be repaired and should ideally be done at the pre-school age [5].

## Conclusions

Poland syndrome is a rare congenital anomaly characterized by the unilateral absence or under-development of pectoralis major and associated with the involvement of adjacent shoulder muscles, rib abnormalities, dextrocardia, and ipsilateral hand deformities. It can also be associated with Sprengel deformity, Klippel-Feil syndrome, scoliosis, Moebius syndrome, and visceral anomalies, such as renal agenesis, hernia, and atrial septal defect, and blood dyscrasias like leukemia and lymphoma. Treatment should be individualized depending on age, sex, degree of deformity, and patient’s preference.
